# Effect of Salinity on Growth, Ion Accumulation and Mineral Nutrition of Different Accessions of a Crop Wild Relative Legume Species, *Trifolium fragiferum*

**DOI:** 10.3390/plants11060797

**Published:** 2022-03-17

**Authors:** Astra Jēkabsone, Una Andersone-Ozola, Andis Karlsons, Māris Romanovs, Gederts Ievinsh

**Affiliations:** 1 Department of Plant Physiology, Faculty of Biology, University of Latvia, 1 Jelgavas Str., LV-1004 Rīga, Latvia; astra.jekabsone@lu.lv (A.J.); una.andersone-ozola@lu.lv (U.A.-O.); maris.romanovs@lu.lv (M.R.); 2 Institute of Biology, University of Latvia, 4 Ojāra Vācieša Str., LV-1004 Rīga, Latvia; andis.karlsons@lu.lv

**Keywords:** forage legumes, growth, ions, mineral nutrition, salinity tolerance, strawberry clover

## Abstract

Crop wild relatives represent a valuable resource for the breeding of new crop varieties suitable for sustainable productivity in conditions of climate change. The aim of the present study was to assess salt tolerance of several wild accessions of *T. fragiferum* from habitats with different salinity levels in controlled conditions. Decrease of plant biomass and changes in partitioning between different organs was a characteristic response of plants with increasing substrate salinity, but these responses were genotype-specific. In several accessions, salinity stimulated reproductive development. The major differences in salinity responses between various *T. fragiferum* genotypes were at the level of dry biomass accumulation as well as water accumulation in plant tissues, resulting in relatively more similar effect on fresh mass. Na^+^ and Cl^−^ accumulation capacity were organ-specific, with leaf petioles accumulating more, followed by leaf blades and stolons. Responses of mineral nutrition clearly were both genotype- and organ-specific, but several elements showed a relatively general pattern, such as increase in Zn concentration in all plant parts, and decrease in Ca and Mg concentration. Alterations in mineralome possibly reflect a reprogramming of the metabolism to adapt to changes in growth, morphology and ion accumulation resulting from effect of NaCl. High intraspecies morphological and physiological variability in responses of *T. fragiferum* accessions to salinity allow to describe them as ecotypes.

## 1. Introduction

Only relatively recently a concept of crop wild relative (CWR) plant species has been established [1,2] and it has been verified that CWRs represent extremely valuable potential resource for breeding new crop varieties [3]. In a light of global climate changes, with predicted increase in severity of deviations in environmental constraints, cultivated plants need to possess higher adaptive plasticity towards a range of suboptimal abiotic factors, allowing them to maintain productivity in highly heterogeneous conditions [4]. In this respect, CWRs can be used as a source of resilience-associated characteristics due to generally higher abiotic stress tolerance [3,5].

Soil salinization represents one of the most urgent problems in agriculture [6], and its negative effects on crop productivity are anticipated to become more severe on a background of global climate changes [7]. Because of their symbiosis with nitrogen-fixing rhizobacteria, salt-tolerant forage legume species are especially important for saline marginal lands with characteristically low response to nitrogen fertilizers [8]. Several species from genus *Trifolium* are commonly used in permanent temperate grasslands, and *Trifolium pratensis* and *Trifolium repens* are considered as especially important CWRs in Europe [9]. *Trifolium fragiferum* is a perennial stoloniferous clover species native to Europe, Mediterranean region, Middle East and West Asia [10]. Due to the relative rarity of *T. fragiferum* in Northern Europe, *T. fragiferum* is legally protected in several countries, including Latvia [11]. While not used commercially in Europe, *T. fragiferum* has been cultivated as forage legume crop in temperate grasslands of Australia and USA [12,13]. The first successful cultivar of *T. fragiferum*, ‘Palestine’, had been developed in Australia from a material collected near the Dead Sea and used commercially since 1938 [13].

Resilience of *T. fragiferum* has been associated both with clonal type of growth as well as high abiotic stress tolerance of the species. Monopodially branching creeping shoots (stolons) have an ability to fom roots at the nodes [14]. Together with moderate tolerance to soil salinity and alkalinity, *T. fragiferum* also has good flooding tolerance [15], an ability to withstand continuous grazing [16] and repeated trampling [17]. Potential suitability of different wild accessions of *T. fragiferum* to saline conditions is of special interest, as it was established that a wide genetic diversity exists within a species in respect to degree of salt tolerance [18]. As in the Northern Europe *T. fragiferum* is exclusively associated with an endangered habitat ‘Baltic coastal meadow’ [19], experimental assessment of populations around the Baltic Sea seems to be extremely promising in order to find highly salt-tolerant physiological types of the species useful for further breeding purposes.

Aspects of plant mineral nutrition have been often related with their salinity tolerance, as mineral imbalance resulting due to salinity treatment can be considered as one of indications of general metabolic disorder [20]. More specifically, Na^+^ accumulation in plant tissues due to increased substrate NaCl can affect their K^+^ status, and consequently, result in disruption of cellular functions. The strategy of active salt exclusion from photosynthetic tissues is a possible adaptive mechanism for salt-tolerant glycophytes and monocotyledonous halophytes [21]. For other halophytes, vacuolar sequestration of Na^+^ and Cl^−^ and maintenance of stable cytosolic K^+^, as an avoidance mechanism, together with accumulation of nonionic osmolytes, leads to stabilization of osmotic homeostasis [22,23], concomitantly with readjusting of cellular mineral balance according to the needs of salinity-altered metabolism [24]. Therefore, assessment of salt-induced changes in mineral element concentration in plant tissues can provide information on adaptive cellular responses possibly related to differences in the degree of salinity tolerance.

Evaluation of local diversity of CWRs is an important constituent in a system of sustainable use of biological resources [25]. A number of geographically-isolated micropopulations of *T. fragiferum* associated with natural water reservoirs have been identified in Latvia recently [26]. Tolerance of several of these accessions of *T. fragiferum* against soil waterlogging and flooding, trampling as well as cutting have been evaluated [17]. All accessions appeared to be relatively tolerant to these factors, but accession-specific differences found suggested existence of different physiological types. The aim of the present study was to assess the salinity tolerance of several wild Latvian accessions of *T. fragiferum* from habitats with different salinity levels in comparison to commercial cultivar ‘Palestine’ as well as *T. fragiferum* accession from a relatively highly saline meadow in Bornholm, Denmark. It was hypothesized that the accessions from habitats with higher soil salinity would be more salinity tolerant in controlled conditions.

## 2. Results

**Figure 1:**
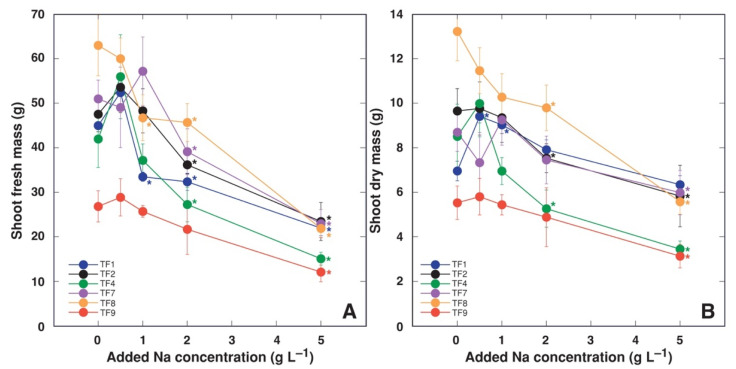
Effect of increasing substrate salinity on shoot fresh mass (**A**) and shoot dry mass (**B**) of *Trifolium fragiferum* plants of different accessions. Asterisks of respective color indicate statistically significant differences from control (*p* < 0.05).

**Figure 2:**
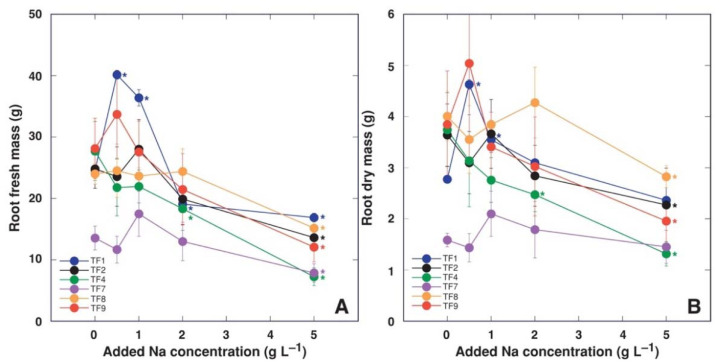
Effect of increasing substrate salinity on root fresh mass (**A**) and root dry mass (**B**) of *Trifolium fragiferum* plants of different accessions. Asterisks of respective color indicate statistically significant differences from control (*p* < 0.05).

Morphological differences were observed between control plants of different *T. fagiferum* accessions during cultivation and at the end of the experiment. Thus, plants of accession TF9 had the lowest shoot biomass (Figure 1) but the highest number of stolons and leaves (Table 1). Plants of TF8 (cv. ‘Palestine’) had the highest shoot biomass in control conditions (Figure 1) and the lowest number of stolons (Table 1). In addition, the longest total length of stolons was evident for plants of accession TF7, but the shortest was seen for accession TF4 (Table 1). Biomass of roots for control plants showed less variance between different accessions, significantly lower values of fresh and dry mass was evident only for TF7 (Figure 2).

When treated with low level of NaCl, several accessions showed a tendency for increased mass of shoots (Figure 1), but only for TF1 dry mass of shoots significantly increased at 0.5 and 1 g L^−1^ (Figure 1B). Plants of accessions TF1 and TF8 exhibited significant decrease of shoot fresh mass already at 1 g L^−1^ Na, but all accessions except TF9 had significant decrease of shoot fresh mass at 2 g L^−1^ Na (Figure 1A). In respect to shoot dry mass, significant decrease for TF2, TF4 and TF8 was evident already at 2 g L^−1^ Na^+^, but all accessions except TF1 exhibited significant biomass reduction at 5 g L^−1^ (Figure 1B). Both fresh and dry mass of roots was significantly stimulated at low Na^+^ concentration only for TF1 (Figure 2). While fresh mass of roots significantly decreased for all accessions at 5 g L^−1^ Na (Figure 2A), root dry mass of TF1 and TF7 did not decrease at this concentration of Na^+^ (Figure 2B). There was no stimulative effect of low Na^+^ on number of stolons, total length of stolons, and number of leaves for any of accessions of *T. fragiferum* (Table 1). These parameters were significantly reduced by 5 g L^−1^ Na^+^ treatment for all accessions, or even at lower concentrations for several accessions.

**Figure 3:**
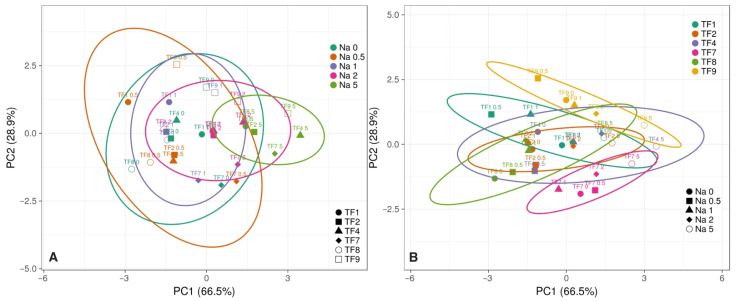
Principal component analysis on effect of increasing substrate salinity on shoot and root fresh mass and dry mass of *Trifolium fragiferum* plants of different accessions. (**A**), grouping according salinity levels; (**B**), grouping according accessions. Prediction ellipses are such that with probability 0.95, a new observation from the same group will fall inside the ellipse. Unit variance scaling was applied to rows; singular value decomposition with imputation was used to calculate principal components. X and Y axes show principal component one and principal component two that explain 66.5% and 28.9% of the total variance, respectively.

**Figure 4:**
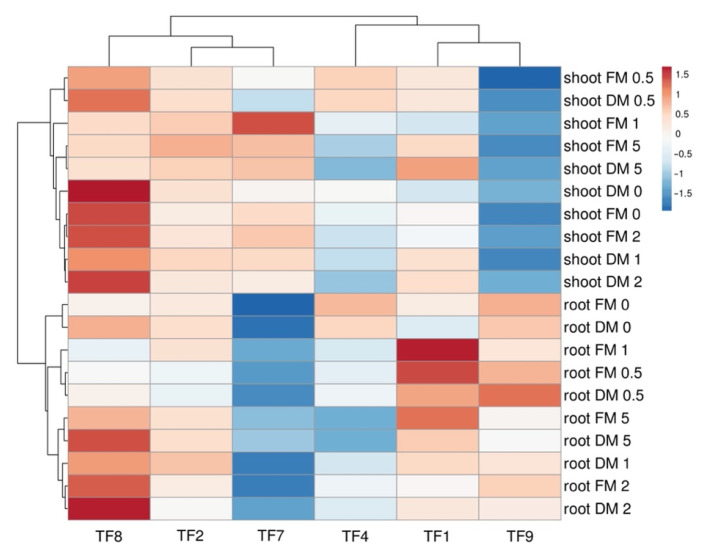
Generated heat map and cluster analysis on effect of increasing substrate salinity on shoot and root fresh mass and dry mass of *Trifolium fragiferum* plants of different accessions. Hierarchical clusters were generated by average linkage method with correlation distance. Color scale shows relative intensity of normalized parameter values. FM, fresh mass; DM, dry mass.

**Figure 5:**
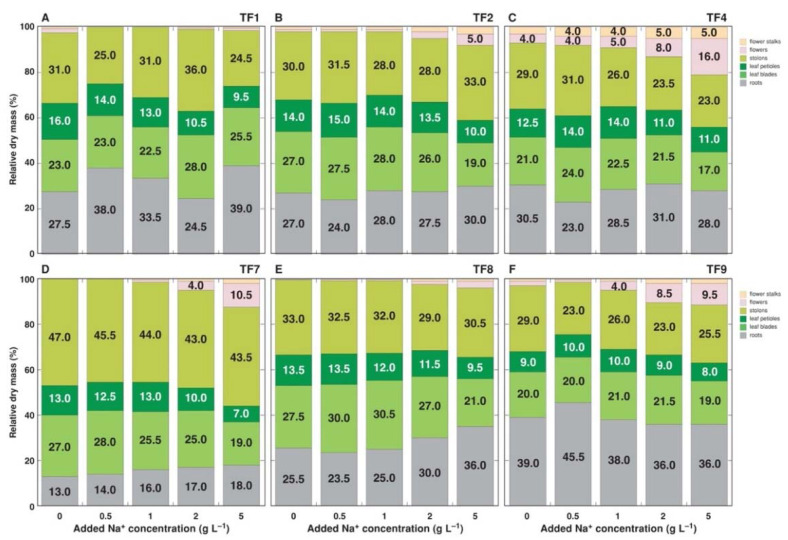
Changes in biomass partitioning in *Trifolium fragiferum* plants of different accessions due to increasing substrate salinity. (**A**), accession TF1; (**B**), accession TF2; (**C**), accession TF4; (**D**), accession TF7; (**E**), accession TF8; (**F**), accession TF9.

It appeared that increasing NaCl concentration in substrate reduced initial differences in fresh and dry mass of shoots and roots between accessions, and this phenomenon was clearly evident by the results of multivariate analysis (Figure 3A). The largest differences between the genotypes in respect to biomass accumulation in roots and shoots were between TF7 and TF9 (Figure 3B), but the largest similarity between TF2 and TF7, and TF1 and TF9 (Figure 4). Analysis of changes in biomass partition also confirmed genotype specificity of salinity effects (Figure 5). Thus, increasing salinity stimulated generative reproduction, and this effect increased in an order TF1 < TF8 < TF2 < TF7 < TF9 < TF4, but partition to roots was enhanced in TF7 and TF8.

**Figure 6:**
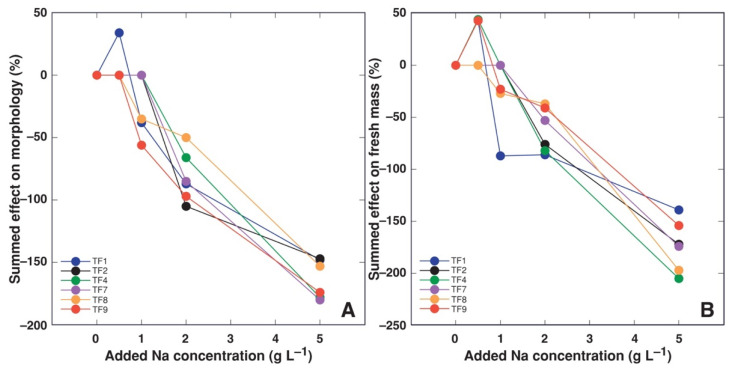

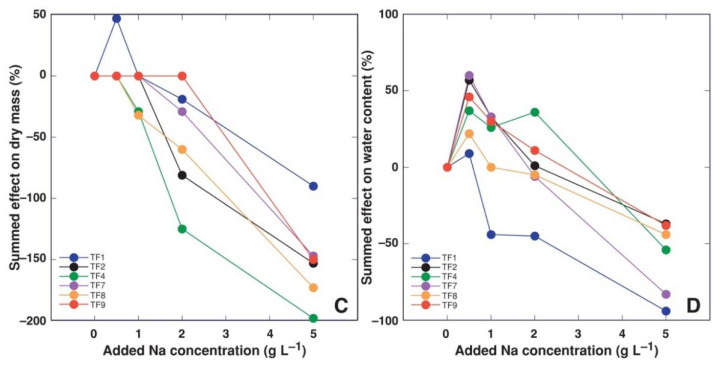
Summed relative effect of increasing substrate salinity on morphology (**A**), fresh mass of plant parts (**B**), dry mass of plant parts (**C**) and water content in plant parts (**D**) of *Trifolium fragiferum* plants of different accessions. Only statistically significant effects are taken into account.

Analysis of summed relative effect of salinity revealed that the major differences in salinity responses between various *T. fragiferum* genotypes were at the level of dry biomass accumulation (Figure 6C) as well as water accumulation in plant tissues (Figure 6D), resulting in relatively more similar effect on fresh mass (Figure 6A). Moreover, effect on morphological indices (number of stolons and leaves, as well as stolon length) was rather consistent between different genotypes (Figure 6B).

**Figure 7:**
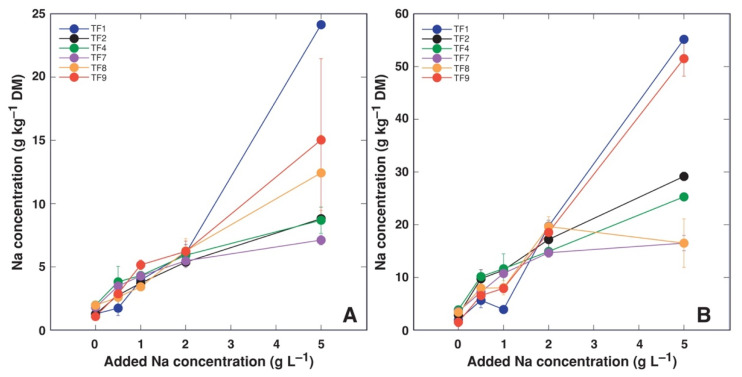

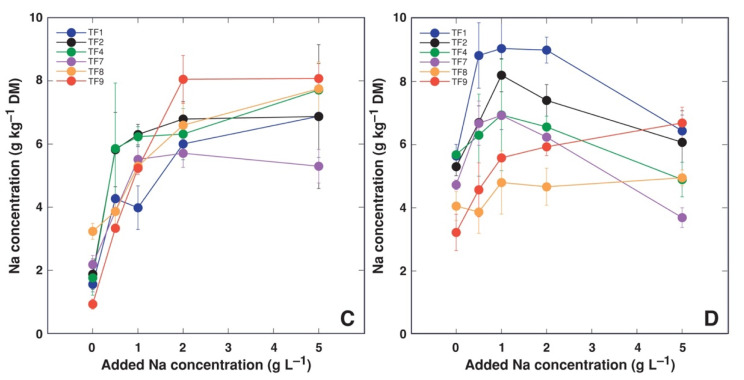
Effect of increasing substrate salinity on Na^+^ accumulation in leaf blades (**A**), leaf petioles (**B**), stolons (**C**) and roots (**D**) of *Trifolium fragiferum* plants of different accessions.

**Figure 8:**
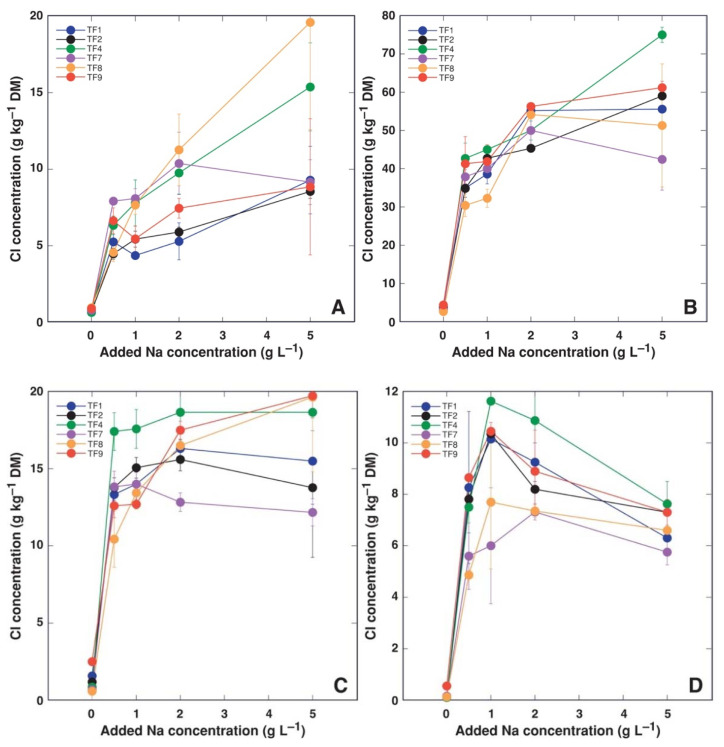
Effect of increasing substrate salinity on Cl^−^ accumulation in leaf blades (**A**), leaf petioles (**B**), stolons (**C**) and roots (**D**) of *Trifolium fragiferum* plants of different accessions.

**Figure 9:**
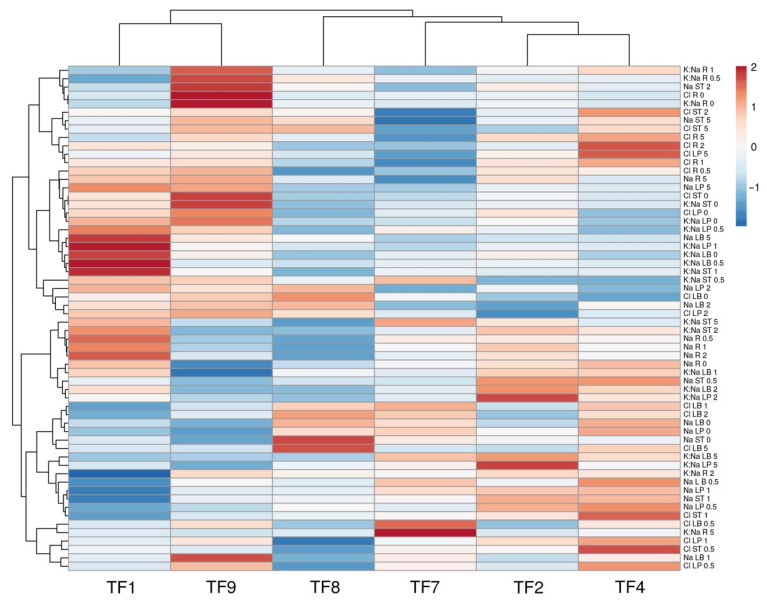
Generated heat map and cluster analysis on effect of increasing substrate salinity on Na^+^ and Cl^−^ accumulation and K:Na ratio in different parts of *Trifolium fragiferum* plants of different accessions. Hierarchical clusters were generated by average linkage method with correlation distance. Color scale shows relative intensity of normalized parameter values. LB, leaf blades; LP, leaf petioles; ST, stolons; R, roots.

In general, Na^+^ accumulation capacity was organ-specific, with leaf petioles accumulating more Na^+^, followed by leaf blades (Figure 7). At low salinity, there were no significant differences in accumulation of Na^+^ in leaf blades (Figure 7A), leaf petioles (Figure 7B) and stolons (Figure 7C), only at the highest salinity (5 g Na^+^ L^−1^) plants from most saline habitats (TF1 and TF9) tended to accumulate more Na^+^ in leaves. In contrast, differences in trend of Na^+^ accumulation in dependence on increasing salinity were evident in plant roots (Figure 7). Accumulation capacity for Cl^−^ was also highest in leaf petioles, followed by stolons and leaf blades (Figure 8). Response of Cl^−^ accumulation was saturable at low substrate NaCl concentration, especially, for stolons and roots. Multivariate analysis of ion accumulation characteristics in plant parts revealed that salinity effects were rather genotype-specific, with closer similarity between TF2 and TF4, as well as TF1 and TF9 (Figure 9).

**Figure 10:**
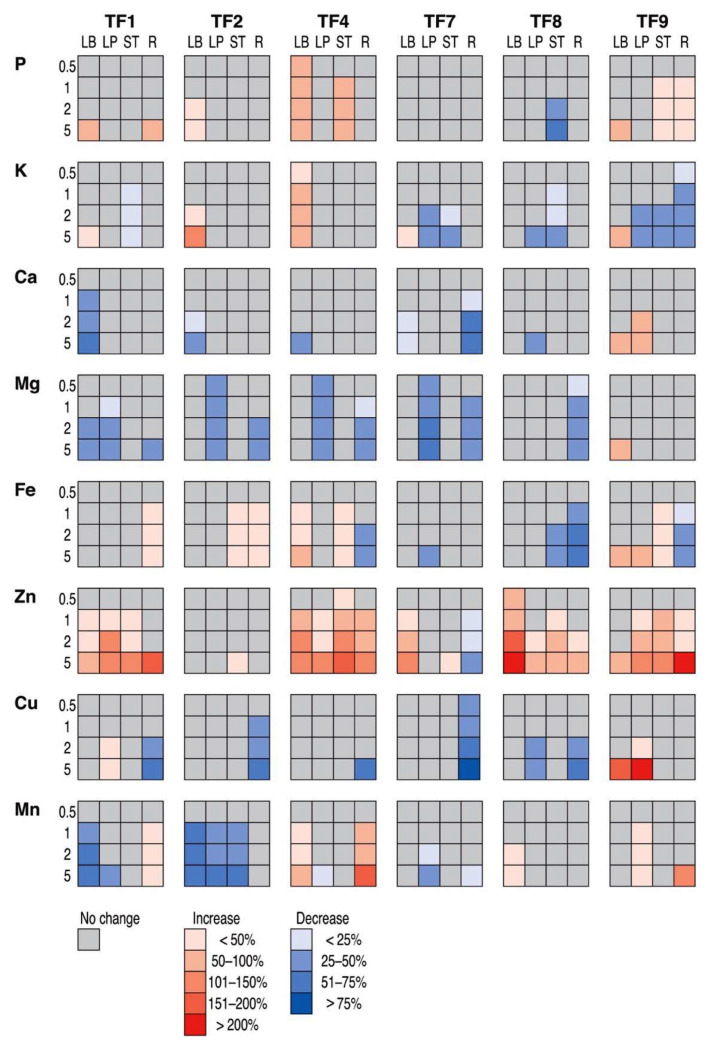
Relative effect of increasing substrate salinity on mineral element concentrations in leaf blades (LB), leaf petioles (LP), stolons (ST) and roots (R) of *Trifolium fragiferum* plants of different accessions. Numbers on the left side indicate added Na+ concentration (g L^−1^). Only statistically significant effects are taken into account.

**Figure 11:**
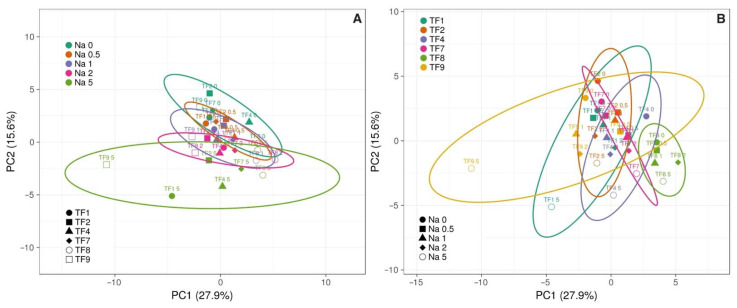
Principal component analysis on effect of increasing substrate salinity on mineral nutrient concentration in different parts of *Trifolium fragiferum* plants of different accessions. (**A**), grouping according salinity levels; (**B**), grouping according accessions. Prediction ellipses are such that with probability 0.95, a new observation from the same group will fall inside the ellipse. Unit variance scaling was applied to rows; singular value decomposition with imputation was used to calculate principal components. X and Y axes show principal component one and principal component two that explain 27.9% and 15.6% of the total variance, respectively.

Effect of salinity on mineral nutrition was evaluated by comparison of relative effect of increasing substrate salinity in various plant parts for different accessions (Figure 10). The responses clearly were both genotype- and organ-specific, but some general trends were evident. Thus, Zn concentration mostly increased in all plant parts for all genotypes except TF2 and TF7, but Ca and Mg concentration decreased, except TF9. Effects on macronutrient P and K, as well as micronutrient Fe, Cu and Mn concentration were rather controversial. According to principal component analysis, diversity in mineral nutrient concentration increased with increasing salinity (Figure 11), and each genotype had rather unique mineral element response trend in different plant parts caused by salinity gradient (data not shown).

## 3. Discussion

### 3.1. Comparison of Salinity Tolerance

*T. fragiferum* as a halophytic species has been included in the eHALOPH database (https://www.sussex.ac.uk/affiliates/halophytes/index.php, accessed on 2 February 2022) as based on the main criterion, tolerance to substrate EC at least 7.8 dS m^−1^ (equivalent to 7.8 mS cm^−1^). This assumption was confirmed also by the results of the present study, with all accessions being able to grow and reproduce at 5 g Na L^−1^ with substrate EC_1:5_ reaching 9.77 mS cm^−1^ (3602 mS m^−1^ by a sensor measurement). Similar salinity level was recorded also in a natural habitat of TF9 on the island of Bornholm (2749 mS m^−1^). The species even has been defined as obligatory mesohydrohalophile, as based on its presence in salt marsh vegetation in Romania [27], but within the northern part of the distribution range it seems to be specifically associated with habitats near different water reservoirs but not with increased soil salinity [26].

Accessions of *T. fragiferum* compared in the present study were growing on soils with different salinity level (Table 2). It seems to be logical to expect that the accessions from more saline habitats (as TF1 and TF9) would show higher salinity tolerance in identical conditions of the controlled experiment in comparison to the accessions from habitats with low salinity (as TF2, TF4, TF7). However, in order to approve or reject this hypothesis, it should be understood that the degree of tolerance to changes in a particular environmental factors can be compared differently. Plants from different taxonomic groups are usually compared in a relative way, comparing percent changes of certain growth-related indices relative to control plants, in order to eliminate genotype-associated differences between the control plants. According to this approach, *T. fragiferum* plants of accession TF9 were the most tolerant to 2 g L^−1^ Na^+^ treatment, but TF1 plants were the most tolerant to 5 g L^−1^ Na^+^ (Figure 6C). However, in absolute terms, when looking for the accession producing the highest biomass at high salinity, *T. fragiferum* accessions TF1, TF2, TF7 and TF8 produced identically high amount of biomass at 5 g L^−1^ Na^+^, with values for TF4 and TF9 being significantly lower (Figure 1B). Consequently, from a practical point of forage production, relatively sensitive cv. ‘Palestine’ (TF8) still would have higher yield when cultivated in saline soil because of extremely pronounced biomass production ability of control plants, when compared to relatively most tolerant accession TF9 from saline coastal habitat in Bornholm. In this respect, the most promising Latvian accession of *T. fragiferum* was TF1 from a saline wet shore meadow of Lake Liepājas: the accession showed the highest relative tolerance to 5 g L^−1^ Na^+^ and also were among the accessions with the highest absolute biomass production capacity at this salinity level. Accession TF1 was also the only one used in the present study showing significant growth stimulation of both shoots and roots at 0.5 and 1 g L^−1^ Na^+^. Besides, TF1 was also the accession most stable to action of several abiotic factors, with very high tolerance to soil waterlogging and repeated cutting, and high tolerance against trampling [17].

While several studies previously have accessed salinity tolerance of *T. fragiferum* [28,29], direct comparison of the results obtained is rather difficult. The main reason is a lack of information on the precise amount of applied salts during treatments, and/or on final salinity level in substrate, measured either as substrate electrical conductivity or concentration of Na^+^. When decrease in biomass accumulation is viewed as a main indication of a plant’s sensitivity to salinity, a possibility that growth inhibition represents a regulated adaptive response to increased salt concentration is usually forgotten. However, changes in biomass partition within a plant with increase in substrate salinity clearly indicate that this assumption could be correct, as showed also in the present study (Figure 5).

Salinity tolerance under different flooding regimes of *T. fragiferum* cv. ‘Palestine’ has been compared with that of other *Trifolium* species and it was shown that the species is more sensitive to salinity than to flooding [28]. During the study with 95 *T. fragiferum* accessions and five cultivars it was concluded that within the species a wide genetic diversity exists in respect to salinity tolerance [18]. Several accessions, when grown at low salinity, even showed significant growth stimulation of their shoots. In hydroponics, dry biomass of cv. ‘Palestine’ decreased to 43–54% at 160 mM NaCl relative to control, but mixed plant sample from five pooled wild accessions of *T. fragiferum* had relatively better tolerance, with biomass decreasing only to 73% at the same salinity [30]. These results are similar to the ones obtained in the present study. Most importantly, it is evident that intraspecies physiological diversity of salinity responses for *T. fragiferum* exist, even from a relatively restricted territory as in the case of Latvia.

No detailed physiological mechanisms of salinity tolerance/sensitivity have been investigated for any of *Trifolium* species so far. However, some insights were made for moderately salt tolerant species *Trifolium alexandrinum*, showing that excessive accumulation of Na^+^ in leaves together with inability for sequestration in vacuoles have led to inhibition of photosynthesis followed by growth inhibition [31]. Similarly, *T. repens* plants from a population accumulating lower amount of Na^+^ and Cl^−^ in shoots, had higher shoot dry mass and lower dieback rate in saline conditions in comparison to plants from a higher-accumulating population [32]. Consequently, a relationship between Na^+^ and Cl^−^ accumulation in plant shoot tissues and their salinity tolerance can be proposed.

### 3.2. Comparison of Ion Accumulation

*T. fragiferum* has been characterized as a species excluding Na^+^ from shoots based simply on the fact that other species of the genus accumulated more Na^+^ [30]. Exclusion of Na^+^ and Cl^−^ from shoot tissues is considered as a characteristic important for salinity tolerance, but it seems to be relevant only for glycophytes [33]. In contrast, halophytic plants are able to use Na^+^ for osmotic adjustment, at least, in vacuoles [34].

In the present study, both *T. fagiferum* accessions from habitats with the highest salinity (TF1 and TF9) had the highest Na^+^ accumulation potential in leaf blades and petioles (Figure 7A,B). In leaf petioles of both accessions, Na^+^ concentration reached >50 g kg^−1^. Interestingly, in natural conditions, TF1 plants were not among the accessions showing highest levels of Na^+^ accumulation, with Na^+^ concentration in petioles reaching only 14 g kg^−1^, while that in TF2 was 21 g kg^−1^ [26]. However, it is important that at low to moderate salinity there were no differences in Na^+^ accumulation potential in leaves between different accessions in the present study in controlled conditions, but these were pronounced in stolons and, especially, in roots. Accumulation potential for the two ions in stolons and roots was less, especially, at high salinity. In comparison, Na^+^ accumulation potential in shoots of *T. repens* was up to 38 g kg^−1^ Na^+^ and 85 g kg^−1^ Cl^−^, while in roots it was only 5 g kg^−1^ Na^+^ and 8 g kg^−1^ Cl^−^ [35]. Consequently, relatively better salinity tolerance of *T. fragiferum* is not associated with differences in accumulation of Na^+^ and Cl^−^. This was also not the case when tolerance of individual *T. fragiferum* accessions were considered: the two relatively most tolerant accessions, TF1 and TF9, accumulated higher Na^+^ concentration only at 5 g L^−1^ Na in leaf petioles, but no such relationship was evident in stolons and roots, or for Cl^−^ accumulation in all plant parts.

Plants of cv. ‘Palestine’ accumulated 1.63–1.86 mmol Na^+^ and 1.56–1.60 mmol Cl^−^ per g DM in shoots (equivalent to 37.5–42.8 g kg^−1^ Na^+^ and 55.4–56.8 g kg^−1^ Cl^−^), but concentrations for wild accessions of *T. fragiferum* were 1.12 mmol Na^+^ and 1.32 mmol Cl^−^ per g DM (equivalent to 25.8 g kg^−1^ Na^+^ and 46.9 g kg^−1^ Cl^−^) [30]. Thus, accumulation range of Na^+^ and Cl^−^ at relatively high substrate salinity for different *T. fragiferum* genotypes is relatively similar, but significant differences in the accumulation potential between different plant parts need to be taken into account (Figure 7 and Figure 8).

Increased water accumulation in plant tissues in response to increasing salinity can be viewed as means for dilution of soluble ions concomitantly with stimulation of vacuolar development [36]. It seems that several accessions of *T. fragiferum* employed this mechanism, especially, at low to moderate salinity (Figure 6). The relationship between salinity tolerance and salinity-induced tissue succulence has been shown for a number of species [37,38,39,40]. However, no such relationship was evident for *T. fragiferum*, as relatively salt tolerant accession TF1 showed only relatively little increase of tissue water content in comparison to that in other accessions (Figure 6).

### 3.3. Mineral Nutrition

Disbalance of mineral nutrition has been suggested as one of the deleterious physiological effects in plants due to high salinity [20]. However, generalization of results from mineral nutrition studies of plants under salinity seems to be rather rare in scientific literature. It has been concluded that the main nutrient-related problem during salinity could be related to mineral imbalance as a result of competition of Na^+^ and Cl^−^ with K^+^, Ca^2+^, and NO_3_^−^, but micronutrient concentrations are relatively less affected [20,24,41]. It was concluded that besides rather pronounced effects on K^+^ uptake and distribution, more supported is the idea of negative effect of salinity on Ca^2+^ uptake, pointing to important role of Ca^2+^ in maintenance of mineral homeostasis in saline conditions.

Increase in shoot K concentration is a commonly described tolerance-associated response of glycophytic species to salinity [33], while osmotic functions of K^+^ can be taken over by Na^+^ in halophytic species [42]. Shoot K^+^ concentration decreased from 1.54 mmol g^−1^ DM in control plants of cv. ‘Palestine’ to 1.03 mmol g^−1^ DM in plants cultivated at 160 mM NaCl (equivalent to 60.1 and 40.2 g kg^−1^), and from 1.76 mmol per g in control plants of wild accessions to 1.24 mmol per g at 160 mM NaCl (equivalent to 69.6 and 48.4 g kg^−1^) [31]. However, no organ-specific effects on K^+^ accumulation were considered so far. In the present study K^+^ concentration in leaf blades significantly increased in all accessions except cv. ‘Palestine’ at least at the highest Na^+^ concentration, but decrease in other parts was evident for several accessions (Figure 10). Interestingly, the most pronounced decrease in tissue K^+^ in leaf petioles, stolons and roots was seen with increasing salinity in presumably most salinity-tolerant accession TF9, as well as for TF1 in stolons. This clearly points to ion accumulation features similar to these of halophytes.

In some species K^+^:Na^+^ ratio has been shown as a reliable indicator of salinity tolerance [43]. This has not been the case for *Trifolium* species, as more salinity-tolerant *T. fragiferum* had lower K^+^:Na^+^ ratio as less salinity-tolerant *T. repens* [44]. Also, in the present study, no relationship was found between K^+^:Na^+^ ratio in different organs of *T. fragiferum* plants from various accessions and their salinity tolerance.

In taxonomically and morphologically related species, *T. repens*, increasing salinity intensity induced a concomitant increase in concentration of several micronutrients in plant roots, including Fe, Mn, and Zn [35]. Proportional increase of Mn concentration with salinity has been described in another legume species, *Melilotus segetalis* [45]. Increase in concentration of Zn as a result of salinity was very noticeable, but with certain genotype-specific differences. This clearly suggests involvement in adaptations to salinity, and usually increased Zn concentration has been associated with its involvement as a component in defense-related proteins, as zinc finger proteins or antioxidative enzyme CuZn-superoxide dismutase [46,47]. It is confirmed that enhanced activity of enzymatic antioxidative system is a prerequisite for salinity tolerance [48].

As based on both literature analysis as well as the results of the present study, it seems that particular salinity-induced changes in the concentration of individual mineral elements are extremely variable even between taxonomically related species and within the species. Thus, for two closely related species, *Limonium perezii* and *Limonium sinuatum*, shoot Mg concentration either increased or decreased, respectively, as a result of increasing salinity [49]. This means that interpretation of results from mineral nutrition studies searching for general salinity effects using single species or only a few different species should be done with caution.

In the present study with *T. fragiferum*, the observed salinity-dependent changes in mineral nutrients were both genotype- and plant part-specific, with no clearly evident relationship with relative salinity tolerance of the genotype (Figure 9). An interesting general characteristic response of mineral nutrition was an increase in the diversity of distribution of concentrations of mineral nutrients at high salinity (Figure 11). Due to relatively high tolerance of all tested accessions to salinity, it seems that the recorded characteristic and genotype-specific changes in concentration of mineral nutrients in plant tissues reflect adaptive responses related to maintenance of metabolic homeostasis in plants growing in saline soil. Similarly, reallocation of mineral nutrients in all plant organs is thought to represent a whole-plant adaptive response [45]. Genotype-specific response of mineral nutrition to increased salinity has been noted also for various salt-adapted halophyte species [50].

### 3.4. Limitations and Benefits of the Experimental System and Future Perspectives

A study similar to this has been performed with another legume species, *Medicago ciliaris*, using seed material from seven spontaneous local populations in Tunisia, and it was concluded that this type of studies represent an efficient approach to find salt response-related genetic variation [51]. However, the problem of data interpretation in studies with legume model species is related to their symbiotic relationship with N_2_-fixing bacteria. It has been shown that rhizobial symbiosis not only provides additional nitrogen substances for plant’s needs but also affects tolerance to unfavorable environmental conditions, possibly through upregulation of defense-associated genes [52,53]. Consequently, different plant responses can be obtained in experiments using asymbiotic plants vs spontaneous establishment of rhizobial symbiosis vs. plants inoculated with efficient symbionts. Thus, active rhizobial symbiosis improved salinity tolerance of *Medicago sativa* plants through increased osmotic adjustment and enzymatic antioxidative capacity [54]. Similar findings have been described also for *Medicago truncatula* [55]. It has been also established that presence of rhizobial symbiosis modulates interaction between *T. fragiferum* and *T. repens* on the background of increased substrate salinity [44]. In the present study, to avoid possible problems with inadequate or/and inefficient rhizobial symbiosis when comparing plant accessions from various sites, *T. fragiferum* plants were cultivated asymbiotically. Our further studies have shown that different *T. fragiferum* accessions have highly variable degree of growth-dependence on presence of their native rhizobia (Jēkabsone et al. unpublished results), therefore, it is intended to perform future experiments on salinity responses in different genotypes of *T. fragiferum*, using their native symbiotic bacterial strains.

The novel aspects revealed by the present study concern genotype-specific effects of increased substrate salinity on biomass partitioning as well as Na^+^ and Cl^−^ accumulation capacity between different organs of *T. fragiferum* plants from various accessions. However, salinity tolerance in plants is clearly multigenic in nature [56]. Thus, ability for sustaining ion homeostasis (including ion compartmentation), osmotic protection and antioxidative defense are listed among the most important groups of mechanisms in plant salinity tolerance [57]. In respect to osmotic adjustment under salinity, in the present study, an emphasis was put on inorganic constituents, Na^+^ and K^+^. However, nonionic osmotically active substances are well known for their role in maintenance of osmotic balance in plants under saline conditions, and corresponding scientific evidence has been provided from studies both in natural and controlled conditions [58,59,60]. Therefore, it can be proposed that accumulation of compatible osmolytes is an important constituent of salinity responses also in *T. fragiferum* plants.

At mild or moderate salinity, induction of antioxidative enzymes is an important aspect of salinity tolerance, as shown for *Beta vulgaris* spp. *vulgaris* [61]. Also, higher capacity of enzymatic antioxidative system in salt-tolerant rice landraces has been shown [43]. Our previous results have indicated that decrease of peroxidase activity in leaves of *T. fragiferum* at increased substrate salinity is a good indicator of relative salinity tolerance, but this effect seemed to be associated with salinity-induced increase in tissue water content [44]. Future studies aimed at dissecting molecular mechanisms of salinity tolerance in different *T. fragiferum* accessions clearly need to focus on enzymatic antioxidative defense system and physiological indicators of tissue integrity.

## 4. Materials and Methods

### 4.1. Plant Material

**Figure 12:**
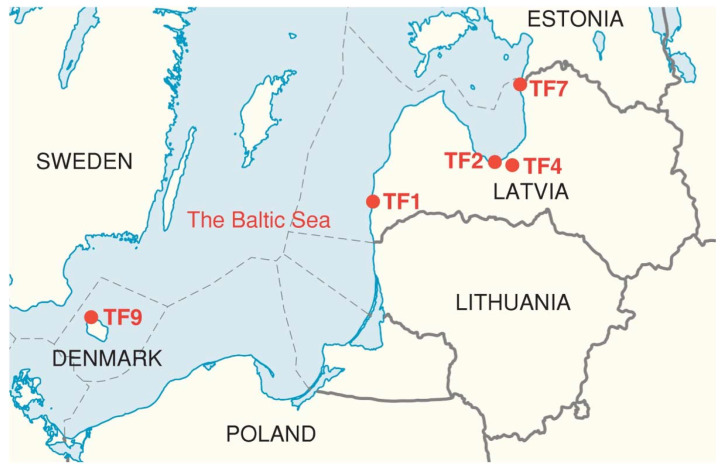
Map of the southern and central Baltic Sea region with *Trifolium fragiferum* accessions used in the present study.

Seeds of *Trifolium fragiferum* from four accessions in Latvia (TF1, TF2, TF4, and TF7) as well as one accession from the island of Bornholm (TF9), from habitats with different salinity levels, were used in the present study (Table 2, Figure 12). *T. fragiferum* cv. ‘Palestine’ (TF8), obtained from Sheffield’s Seeds Company (Locke, NY, USA) was used for comparison.

### 4.2. Cultivation Conditions and Treatments

Plants were cultivated in asymbiotic conditions of soil culture in an automated greenhouse. All details of plant establishment and cultivation were as described previously [17]. Fully acclimatized four week-old plants were randomly divided into five treatments, five plants per treatment as biological replicates. Respective plants were treated with NaCl once a week, adding 1.27 or 2.54 g NaCl dissolved in 200 mL deionized water per container with 1 L of soil substrate until final concentration was reached within five weeks (Table 3). After achieving full treatment, soil electrical conductivity (EC) was measured in containers with HH2 meter equipped with WET-2 sensor (Delta-T Devices, Burwell, UK), and in 1:5 (v/v) soil suspension in deionized water following 15 min incubation with LAQUAtwin conductivity meter B-771 (Horiba Scientific, Kyoto, Japan). Plants were cultivated for additional three weeks then the experiment was terminated.

### 4.3. Measurements

Plants were individually separated in different parts (roots, stolons, leaf petioles, leaf blades, flower stalks, inflorescences). Stolons, leaves and inflorescences were counted, and the length of individual stolons was measured. Plant material was weighed separately before and after drying in an oven at 60 °C for 72 h. Water content was calculated as g H_2_O per g dry mass.

Mineral element analysis in dry-ashed plant material was performed as described previously [26]. After mineralization of the plant samples and dissolving the mineral fraction in either 3% HCl (P, K, Ca, Mg, Fe, Mn, Zn, Cu, Na) or deionized water (Cl), chemical analyses were done using the following methods: the levels of K, Ca, Mg, Fe, Cu, Zn and Mn were estimated by microwave plasma atomic emission spectrometer (MP-AES) Agilent 4200, these of P were analyzed by the colorimetry with ammonium molybdate in an acid-reduced medium using a spectrophotometer Jenway 6300, but values of Cl were obtained by AgNO_3_ titration using distilled water extraction of plant ash. All analyses were performed in triplicate, using representative tissue samples from individual biological replicates.

### 4.4. Data Analysis

As flower-related characteristics were rather variable between individual plants, they were used only for calculation of total shoot biomass as well as for establishment of biomass partitioning [17]. The relative effect of salinity was expressed as percent changes of the parameter in comparison to the respective control plants. Comparison of the relative effect of treatments between different accessions was performed by means of summed percent changes, separately for morphological parameters (number of leaves and stolons, average and total length of stolons), fresh mass of separate plant parts, and dry mass of separate plant parts, as well as water content in plant parts. The total summed effect was calculated by combining percent effect on morphological parameters, fresh mass and dry mass. Only changes significantly statistically different from control values were taken into account for the calculation of summed effects. Effect of salinity on mineral nutrient concentration was estimated as percent increase of the respective concentration in comparison to control plants, taking into account only statistically significant changes.

Results were analyzed by KaleidaGraph (v. 5.0, Synergy Software, Reading, PA, USA). Statistical significance of differences was evaluated by one-way ANOVA using post-hoc analysis with minimum significant difference. Principal component analysis, heat map generation and cluster analysis were performed by a freely available web program ClustVis (http://biit.cs.ut.ee/clustvis/, accessed on 13 March 2022) [62]. For principal component analysis, prediction ellipses were such that with probability 0.95, a new observation from the same group will fall inside the ellipse. Unit variance scaling was applied to rows; singular value decomposition with imputation was used to calculate principal components. Hierarchical clusters were generated by average linkage method with correlation distance.

## 5. Conclusions

High intraspecies morphological and physiological variability is characteristic for responses of *T. fragiferum* accessions to salinity, allowing them to be described as ecotypes. While increasing salinity results in a decrease in the initial biomass differences between accessions, an expansion of morphological variability and diversity of mineral nutrient concentrations among plant parts in saline conditions is strongly pronounced. Changes in mineralome possibly reflect a reprogramming of the metabolism to adapt accordingly to changes in growth, morphology, and ion accumulation resulting from the direct effect of NaCl.

## Figures and Tables

**Table 1:** Effect of increasing substrate salinity on morphological parameters of *Trifolium fragiferum* plants of different accessions.

Treatment	TF1	TF2	TF4	TF7	TF8	TF9
Number of stolons (n)						
0	19.8 ± 3.0 a	25.0 ± 2.7 a	18.8 ± 2.1 a	26.8 ± 3.6 a	18.0 ± 2.4 a	30.8 ± 5.8 a
0.5	20.9 ± 4.3 a	21.8 ± 3.3 a	17.6 ± 2.6 a	22.6 ± 4.2 a	16.8 ± 1.0 a	28.6 ± 3.1 a
1	18.8 ± 3.7 a	25.8 ± 3.0 a	18.2 ± 1.9 a	37.6 ± 8.0 a	16.8 ± 1.9 a	24.2 ± 2.3 a
2	12.7 ± 5.5 a	16.2 ± 2.0 b	16.2 ± 1.9 a	18.0 ± 2.6 b	18.2 ± 3.0 a	18.4 ± 6.0 b
5	12.1 ± 1.4 b	14.8 ± 3.6 b	10.2 ± 1.3 b	14.0 ± 2.0 b	13.2 ± 1.3 b	14.2 ± 3.8 b
Total stolon length (m)						
0	7.38 ± 0.91 a	7.43 ± 1.00 a	3.92 ± 0.70 a	10.28 ± 1.03 a	5.18 ± 1.16 a	6.35 ± 1.27 a
0.5	6.67 ± 0.81 a	7.10 ± 1.10 a	4.05 ± 0.57 a	8.23 ± 2.14 a	4.74 ± 0.36 a	4.97 ± 0.68 a
1	4.56 ± 0.67 b	6.29 ± 0.83 a	2.87 ± 0.41 a	10.54 ± 2.04 a	3.72 ± 0.64 ab	4.11 ± 0.32 ab
2	3.63 ± 0.93 bc	3.94 ± 0.32 b	1.88 ± 0.30 b	4.88 ± 0.59 b	2.65 ± 0.49 b	2.70 ± 0.99 bc
5	2.12 ± 0.20 c	2.21 ± 0.41 c	0.82 ± 0.09 c	2.43 ± 0.34 c	1.35 ± 0.27 c	1.54 ± 0.35 c
Number of leaves (n)						
0	272 ± 33 a	321 ± 24 a	175 ± 21 a	348 ± 47 a	274 ± 45 a	397 ± 92 a
0.5	366 ± 43 a	346 ± 49 a	204 ± 33 a	314 ± 40 a	241 ± 27 a	464 ± 48 a
1	229 ± 46 a	338 ± 33 a	175 ± 19 a	443 ± 58 a	247 ± 33 a	374 ± 44 a
2	289 ± 78 a	247 ± 16 b	151 ± 14 a	294 ± 25 a	291 ± 40 a	322 ± 61 a
5	168 ± 23 b	206 ± 34 b	82 ± 10 b	153 ± 20 b	133 ± 16 b	214 ± 52 b

Different letters for each parameter within a column indicate statistically significant differences (*p* < 0.05) between treatments for the respective accession.

**Table 2:** Characterization, geographical location of accessions of *Trifolium fragiferum* used in the present study and soil electrical conductivity (EC) at the sites.

Code	Associated Water Reservoir	Habitat	Location	Coordinates	Soil EC (mS m^−1^)
TF1	Lake Liepājas	Salt-affected wet shore meadow	City of Liepāja, Latvia	56°29′29″ N, 21°1′38″ E	380 ± 124
TF2	River Lielupe	Salt-affected shore meadow	City of Jūrmala, Lielupe, River Lielupe Estuary, Latvia	57°0′11″ N, 23°55′56″ E	85 ± 5
TF4	–	Degraded urban land	City of Rīga, Vidzeme Suburb, Latvia	56°57′46″ N, 24°7′2″ E	69 ± 6
TF7	The Gulf of Riga of the Baltic Sea	Dry coastal meadow	Town of Ainaži, Latvia	57°52′8″ N, 24°21′10″ E	65 ± 4
TF8 cv. ‘Palestine’	na	na	na	na	na
TF9	The Baltic Sea	Salt-affected wet coastal meadow	Hammeren, Bornholm, Denmark	55°17′54″ N 14°46′17″ E	2749 ± 209

**Table 3:** Experimental treatments used in the present study. Soil EC (measured in containers with a sensor) and soil suspension EC were analyzed after reaching final treatment concentrations. EC, electrical conductivity.

Treatment	Added Salinity (mM)	Amount of Added NaCl (g)	Concentration of Added Na (g L^−1^)	Soil EC (mS m^−1^)	Soil Suspension (1:5) EC (mS cm^−1^)
Control	0	0	0	71.7 ± 7.3	0.27 ± 0.05
0.5	22	1.27	0.5	210.4 ± 10.0	0.50 ± 0.02
1	44	2.54	1.0	353.8 ± 31.0	1.96 ± 0.17
2	87	5.08	2.0	514.5 ± 36.9	3.04 ± 0.72
5	217	12.70	5.0	3602.2 ± 327.5	9.77 ± 1.18

## Data Availability

All data reported here is available from the authors upon request.
